# Evaluation of two novel thoracolumbar trauma classification systems

**DOI:** 10.4103/0019-5413.36995

**Published:** 2007

**Authors:** Alpesh A Patel, Peter G Whang, Darrel S Brodke, Amit Agarwal, Joseph Hong, Carmella Fernandez, Alexander R Vaccaro

**Affiliations:** University of Utah, Departments of Orthopaedic Surgery and Neurosurgery, Salt Lake City, Utah, USA; * Yale University School of Medicine, Department of Orthopaedics and Rehabilitation, New Haven, CT, USA; ** Panorama Orthopedics and Spine, Golden, CO, USA; *** Thomas Jefferson University, Department of Orthopedic Surgery, Philadelphia, PA, USA

**Keywords:** Injury classification, injury severity score, spinal cord injury, thoracolumbar dislocation, thoracolumbar fracture

## Abstract

**Background:**

Despite numerous attempts at classifying thoracolumbar spinal injuries, there remains no consensus on a single unifying algorithm of management. The ideal system should provide diagnostic and prognostic information, exhibit adequate reliability and validity and be easily applicable to clinical practice. The purpose of this study is to assess the reliability and validity of two novel classification systems for thoracolumbar fractures – the Thoracolumbar Injury Severity Score (TLISS) and the Thoracolumbar Injury Classification and Severity Score (TLICS) – and also to discuss potential efforts towards research in the future.

**Matereials and Methods:**

Seventy-one patients with thoracolumbar fractures were prospectively assessed by surgeons with different levels of training and experience (attending orthopedic surgeon, attending neurosurgeon, spine fellows, senior level and junior level residents) at a single institution. Plain radiographs, CT and MRI imaging were used to classify these injuries using the TLISS system. Seven months later, 25 consecutive injuries were prospectively assessed with the TLISS and TLICS systems. Unweighted Cohen's kappa coefficients and Spearman's correlation values were calculated to assess inter-observer reliability and validity at each point in time.

**Results:**

For both the TLISS and TLICS algorithms, the inter-rater kappa statistics for all of the subgroups demonstrated moderate-to-substantial reliability (0.45-0.74), although there were no significant differences among the shared subgroups. The kappa score of the TLISS system was greater than that of the TLICS system for injury mechanism/ morphology. Correlation values were also greater across all subgroups (*P* ≤0.01). Statistically significant improvements in TLISS inter-observer reliability were observed across all TLISS fields (*P* <0.05). The TLISS and TLICS schemes both demonstrated excellent validity.

**Conclusion:**

The TLISS and TLICS scales both exhibited substantial reliability and validity. However, the TLISS system displayed greater inter-observer correlation than did the TLICS and demonstrated significant improvements in reliability over time.

Analogous to the goals of orthopedic traumatologists, spinal physicians strive to prevent deformity progression and associated longterm chronic pain issues and loss of function. Every effort is made to stop the emergence or progression of a neurologic deficit or, where applicable, to promote neurological recovery. The enduring goals are to improve the patient's comfort and locomotion. Classification systems may facilitate the pursuit of these goals.

These systems may be descriptive, mechanistic or based on a multitude of factors including injury severity. Since the work of Bohler in 1929, a number of classification systems have been developed: simple descriptions of the radiographic appearance of an injury, those that attempt to infer the mechanism of injury and systems that attempt to be inclusive of all injury types and subtypes.^1^–^3^

Although multiple classification systems have been produced, there is currently no clear consensus regarding the optimal system for characterizing thoracolumbar fractures. An ideal system must be simple and reproducible based upon commonly identified clinical and radiographic parameters. Current systems are either excessively convoluted, with an impractical number of variables; or are too simple, lacking sufficient detail to provide clinically relevant information. These limitations have yielded classification systems that are difficult to implement, have been shown to possess insufficient validity and reproducibility and have not been widely popular.^4^–^7^

Two novel classification systems have been described: the Thoracolumbar Injury Severity Score (TLISS) and the Thoracolumbar Injury Classification and Severity Score (TLICS).^8^,^9^ The TLISS system defines three primary variables for spinal trauma: 1) mechanism of injury, 2) integrity of the posterior ligamentous complex and 3) the neurological status of the patient [Table 1].^8^ Due to concerns regarding the subjective nature of injury mechanism, the TLICS was subsequently described to include injury morphology in addition to posterior ligamentous integrity and neurological status [Table 2].^9^ The purpose of this manuscript is to describe the reliability and validity of the TLISS and TLICS systems and to discuss future research efforts in thoracolumbar classification systems.

**Table 1:** Thoracolumbar injury severity score illustrating three major categories: mechanism of injury, neurological status and posterior ligamentous complex with associated grading points

Mechanism type	Qualifier	Points
Compression	None	1
Compression	Lateral Angulation >15°	1
Compression	Burst	1
Translational/rotational		3
Distraction		4
**Neurologic involvement**	**Qualifier**	**Points**

Intact		0
Nerve root		2
Cord, conus medullaris	Incomplete	3
	Complete	2
Cauda equina		3
**Posterior ligamentous**		**Points**

Complex		
Intact		0
Injury suspected/Indeterminate		2
Injured		3

**Table 2:** Thoracolumbar injury classification and severity score illustrating three major categories: injury morphology, neurologic status and posterior ligamentous complex with associated grading points

Morphology	Qualifier	Points
Compression	None	1
	Burst	+1
Translational/rotational		3
Distraction		4
**Neurologic Status**	**Qualifier**	**Points**

Intact		0
Nerve root		2
Cord, conus medullaris	Incomplete	3
	Complete	2
Cauda equina		3
**Posterior ligamentous complex**		**Points**

Intact		0
Injury suspected/indeterminate		2
Injured		3

## MATERIALS AND METHODS

Institutional review board approval was obtained prior to the initiation of the study. Seventy-one clinical cases of thoracolumbar spine trauma were prospectively examined. All patients presenting to a single institution with thoracolumbar spine trauma were consecutively included in the study. Prior to definitive treatment, case descriptions were prepared, including the injury mechanism, the neurological examination (including ASIA score) and radiographic images consisting of plain films, CT and MRI. Utilizing the TLISS system, all patients were independently classified by fellowship-trained orthopedic and neurosurgery attending physicians, spine surgery fellows, as well as senior- and junior-level resident physicians utilizing a scoring sheet describing the classification and injury score. After this initial use, the TLISS was implemented in the routine assessment of every thoracolumbar trauma. Seven months after the initial assessment, 25 consecutive cases were assessed utilizing the TLISS system to assess potential changes in reliability over time.

After the description of the TLICS system, the clinical and radiographic data for the subset of 25 cases were randomly reordered and assessed according to the criteria set forth in the TLICS algorithm. The therapeutic options (operative *vs.* nonoperative) recommended by the two sets of injury scores were then compared to the type of treatment that the patient ultimately received.

All data were analyzed by an independent statistician. All statistics were calculated using SPSS software (SPSS Inc., Chicago, IL). Inter-observer reliability was calculated for individual TLISS and TLICS score components, total score and management suggestion using Cohen's unweighted kappa coefficients and Spearman's rank-order correlation. The category of neurological level was not compared as this data was presented to the reviewers (i.e., ASIA grade, exam findings) and was not subject to independent assessment. Face validity was assessed by comparing TLISS and TLICS treatment recommendations (operative versus nonoperative) with the type of treatment the patient ultimately received.

Data are expressed as kappa (κ) ± asymptotic standard error or Spearman's (r) ± asymptotic standard error. Alpha value was set at 0.05. Spearman's rank order correlations and 95% confidence limits for all kappa coefficients were used to determine the statistical significance of differences between the first and second assessment of reliability. Kappa values range from 1.0 (complete agreement) to 0 (no agreement beyond chance). As described by Landis and Koch, negative scores reflect less than chance agreement; 0.01-0.20, slight agreement; 0.41-0.60, moderate agreement; 0.61-0.80, substantial agreement; and 0.81-0.99, almost perfect agreement.^10^

## RESULTS

The inter-observer reliability of the TLISS as assessed by the Cohen's unweighted kappa value showed significant improvement over the seven-month time period [Table 3]. The inter-rater agreement on sub-scores for mechanism, posterior ligamentous complex (PLC), total TLISS and management improved significantly by both Cohen's kappa and Spearman's correlation values (*P* <0.05).

**Table 3:** Summary of reliability data

	Kappa	Correlation
TLISS mechanism 1	0.256	0.384
TLISS mechanism 2	0.636^1^	0.790^1^,^2^
TLICS morphology	0.626^1^	0.684^1^
TLISS PLC 1	0.202	0.327
TLISS PLC 2	0.520^1^	0.737^1^,^2^
TLICS PLC	0.447^1^	0.616^1^
TLISS total score 1	0.189	0.638
TLISS total score 2	0.509^1^	0.885^2^
TLICS total score	0.455^1^	0.852^1^
TLISS management 1	0.455	0.466
TLISS management 2	0.724^1^	0.902^1^,^2^
TLICS management	0.652^1^	0.813^1^

1 Significantly greater than first TLISS assessment (*P* <0.05),

2 Significantly greater than TLICS value (*P* ≤0.01), TLICS - Thoracolumbar injury classiT cation and severity score, TLISS - Thoracolumbar injury severity score

Inter-observer reliability of the TLICS was statistically greater (*P* <0.05) by both Cohen's unweighted kappa and Spearman's correlation scores compared with the initial TLISS assessment. At the second assessment, TLISS sub-scores of injury mechanism, posterior ligamentous complex and management demonstrated significantly (*P* <0.01) higher scores compared with TLICS sub-scores. Both TLISS and TLICS demonstrated good validity in predicting treatment [Table 4].

**Table 4:** Summary of validity data

	% Correct	Sensitivity	Specificity	PPV	NPV
TLISS	92.7	0.889	0.953	0.930	0.925
TLICS	95.4	0.867	0.971	0.951	0.917

## DISCUSSION

The TLISS and TLICS systems were developed to improve and standardize both the understanding and the communication of spinal trauma. This study demonstrates that the TLISS and the TLICS systems can be utilized with good inter-observer reliability and validity. The results further demonstrate that with repeated education and daily use, the reliability of the classification system can improve significantly. This suggests that the content and structure of systems are both sound and easily incorporated into clinical practice.

Historically, the two most popular means of describing an injury are 1) by inferring a mechanism of injury and 2) simply by describing what is seen on static plain X rays, reconstructed CT scan and possibly magnetic resonance imaging. Several mechanisms may result in similar-appearing fractures, and similar mechanisms may result in differing fracture appearances. As such, classifying injuries mechanistically is done primarily through hypothesis and inference. This leaves one with the ability to describe what is immediately visible on an imaging study: the injury morphology. Unfortunately, there is a lack of uniform nomenclature to describe injuries and a tendency to have different interpretations of similar images. It is hoped that the TLICS system provides a framework for future discussions and that future investigations will help settle the debate on morphometry versus mechanism of injury.

Another dilemma in assessing the structural integrity of the spine is that interpretation of the posterior ligamentous complex (PLC) can be difficult. PLC compromise is obvious in some cases where angulation and translation are apparent on plain film X rays. When injury is not apparent on plain radiography, CT imaging is often useful in demonstrating spinal mal-alignment, as well as facet dislocation or subluxation. MRI is considered the most sensitive modality when assessing the status of spinal soft tissues. However, there is a paucity of surgical studies that have correlated MR findings and actual tissue integrity in trauma patients. Lee has suggested that signal changes on fat-suppressed MR images correlate with actual tissue disruption.^11^ However, such findings have not been correlated with the natural history of nonoperative management.

Advancements in MR technology may be more sensitive and specific to particular tissue disruption and may assist in prognosticating the nature of a spinal injury and the degree of potential instability. Conclusions based on indirect imaging technology will have to be validated in prospective studies where surgical findings are correlated with blinded readings of fat-suppressed or short tau inversion recovery (STIR) weighted MR images following thoracolumbar trauma. Additionally, patients treated nonoperatively will truly assist in understanding the importance of MR findings. These nonoperative patients can be followed to determine the natural history of apparent PLC compromise, both radiographically with respect to deformity progression and clinically with respect to pain and function. These important studies will reveal the most valid radiographic indicators of PLC injury while demonstrating the true contribution of the PLC to stability.

The inclusion of a physical examination parameter, neurological injury, to thoracolumbar injury classification is unique to both the TLISS and TLICS systems. The presence of a neurological injury suggests a higher degree of spinal instability and the need for stabilization to prevent further insult. Additionally, individuals with neurological injury may benefit the most from early mobilization (through operative treatment) in order to maintain muscle mass and minimize post-injury complications (pneumonia, infection, deep venous thrombosis (DVT), etc.). The neurological status of the patient is a critical characteristic of thoracolumbar spinal trauma.

The acceptance of a classification system takes place along a continuum. As demonstrated in this study, reliability of classification systems can change over time. A certain period of practice and usage is therefore necessary in order to truly understand and apply the new system. Physicians may comprehend and agree with the nomenclature and components of a classification system, but may not agree on the weighting of its various components or the assigned severity scores. Cultural or geographic differences may also exist. For example, in some regions, brace immobilization of any duration is frowned upon when surgical options exist. Clinicians in other regions seldom or never apply operative techniques due to financial or technical limitations of the health-care system. Obviously, the indices of successful outcomes are defined differently between these societies, regardless of pain relief and function being equal at longterm followup.

A classifications system, therefore, should be culture or society specific and be given time to be learned by clinicians at all levels of expertise. Only then can the validity of the system be objectively assessed in terms of its ability to prognosticate outcome and guide treatment. Severity scores and their associated treatment guidelines should be geared to the expectations of the patient and physician, as well as the documented outcome measures thought important to the society in which they are implemented. If a culture favors early mobilization, threshold scores should be commensurate with that expectation. This may mean that classification scores for particular injuries are similar across regions, but that the threshold values for different management strategies are shifted. International studies assessing the reliability, content validity and construct validity of an injury classification system at multiple time points following its introduction will allow us to better understand these issues of time-dependant learning and regional variability in classification adoption.

Ultimately, to truly assess the validity of a new means of classifying thoracolumbar injuries, well-designed studies with adequate statistical power must be conducted. This is an obvious hurdle when studying trauma populations, considering the inherent inability to recruit patients into varied treatment groups. Given the infrequent occurrence of specific thoracolumbar injury subtypes, it can take years to achieve the statistical power to draw any conclusions. Moreover, multi-surgeon and multi-center studies are confounded by the varied beliefs among treating physicians as to the optimal treatment method and timing of intervention. Generally, treatment for complex traumatic spinal injuries occurs in regional trauma centers, and the method of treatment is often specific to the institution. Frequently, the professor or head of a spinal surgical unit will convey a strong preference on management strategy following the review of a spinal injury. These sentiments towards treatment may appear to be aggressive in one region and conservative in another geographic locale. It is extremely difficult in this culture to randomize specific injuries to various treatment methods as most surgeons have experience with only a subset of the possible treatment approaches for each injury type. Even when a surgeon or center has familiarity with techniques beyond the preferred approaches at that location, the technical quality is unlikely to equal that at an institution specializing or believing in that method of care. Additionally, a particular center may not have the personnel or resources to perform an investigational technique, making it all the more difficult to randomize to this treatment method.

The concept of clinical equipoise allows for the elimination of selection bias by predefining inclusion and exclusion criteria for a study population. Representatives from different treatment camps collectively analyze a particular injury which qualifies for the study and then decide on the method of treatment. For the cases in which agreement cannot be reached, the patients would be managed as per the admitting hospital's expertise. This nonrandomized approach allows for a valid comparison of treatment alternatives for each specific injury subtype in a prospectively identified, well-matched cohort of patients.

This method of treatment comparison may be the future of spinal trauma research. Most institutions do not see an adequate number of patients with specific injury subtypes in order to enable conducting of a sufficiently powerful single-center study. Furthermore, randomization of trauma patients into treatment groups is often not practical and may be viewed unfavorably by institutional review boards. Most importantly, surgeons are relieved of the ethical burden of possibly administering treatments thought inferior to the institution's traditional philosophy of care.
